# COVID-19 vaccinations are associated with reduced fatality rates: Evidence from cross-county quasi-experiments

**DOI:** 10.7189/jogh.11.05019

**Published:** 2021-07-17

**Authors:** Li-Lin Liang, Hsu-Sung Kuo, Hsiu J Ho, Chun-Ying Wu

**Affiliations:** 1 Department of Business Management, National Sun Yat-sen University, Kaohsiung, Taiwan; 2 Research Center for Epidemic Prevention, National Yang Ming Chiao Tung University, Taipei, Taiwan; 3 Institute of Biomedical Informatics, National Yang Ming Chiao Tung University, Taipei, Taiwan; 4 Division of Translational Research, Taipei Veterans General Hospital, Taipei, Taiwan; 5 Department of Public Health, China Medical University, Taichung, Taiwan

## Abstract

**Background:**

Scientists have demonstrated the efficacy of vaccines against severe acute respiratory syndrome coronavirus 2 in randomized controlled trials. However, the extent to which reductions in COVID-19 case fatality ratio (CFR) are attributable to mass vaccination in the real world remains unclear. This study evaluated the association of COVID-19 vaccine coverage with CFR on a global scale.

**Methods:**

The sample was a longitudinal data set of 90 countries over 25 weeks, from the first week of November 2020 to the third week of April 2021. CFR was measured in deaths per 100 COVID-19 confirmed cases; vaccine coverage was defined as the number of people who received at least one vaccine dose per 10 people in the total population. Data were retrieved from open-access databases, including Our World in Data and the Oxford COVID-19 Government Response Tracker. A country-level random effects model was used; a comprehensive set of variables for country characteristics and nonpharmaceutical interventions were included.

**Results:**

A 10% increase in vaccine coverage was associated with a 7.6% reduction in the CFR (95% confidence interval (CI = -12.6 to -2.7%, *P* = 0.002). This association was stronger in countries with more effective governments (-8.3%; 95% CI = -13.6 to -3.1%, *P* = 0.002) and higher transport infrastructure quality (-8.1%; 95% CI = -13.3 to -2.9%, *P* = 0.002). Moreover, the vaccine coverage was associated with a reduced CFR in a dose-dependent manner. When vaccine coverage achieved 0.8 to 1.6, 1.6 to 3.2 and ≥3.2 per 10 people, the CFR reduced by 12.7% (95 CI = -21.8 to -3.6%, *P* = 0.006), 21.2% (95 CI = -33.9 to -8.5%, *P* = 0.001) and 31.3% (95 CI = -51.5 to -11.0%, *P* = 0.002), respectively as compared with no vaccination.

**Conclusions:**

Our results provide supporting evidence that vaccination is critical to preventing deaths among infected people. Vaccination programmes have yielded significant health benefits in certain countries. However, globally, a large gap remains between observed and achievable fatality reductions. Continuous improvement in vaccine coverage will be critical to transforming efficacious vaccines into desired health outcomes.

The coronavirus disease 2019 (COVID-19) pandemic had infected more than 158 million people and caused more than 3.2 million deaths worldwide as of May 11, 2021. Nonpharmaceutical interventions such as workplace closings, gathering restrictions, public transport closures, and travelling restrictions have demonstrated effectiveness in limiting the spread of COVID-19 [1,2]. However, these polices incur economic and social costs. Vaccines against severe acute respiratory syndrome coronavirus 2 (SARS-CoV-2) are expected to be a critical measure to mitigate the burden of COVID-19 worldwide [3].

Clinical trials of COVID-19 vaccines have focused on the safety of the vaccine, efficacy of infection reduction, severity of resultant diseases, and duration of infectivity [4,5]. The key efficacy endpoint, protection against mortality, is difficult to assess in phase 3 clinical trials [6]. Most importantly, vaccine efficacy data obtained under ideal conditions (eg, randomised controlled trials) may not predict actual vaccine effectiveness under field conditions. Vaccine effectiveness is measured as health benefits attributable to vaccines administered through public health programmes [7]. This distinction represents a large gap in empirical research, with disproportional efforts devoted to COVID-19 vaccine efficacy, leaving vaccine effectiveness understudied, in particular how vaccine programmes affect COVID-19 fatalities in the real world.

Among the few studies that have evaluated COVID-19 vaccine effectiveness, the results primarily relate to Israel’s nationwide vaccination campaigns. Dagan et al. (2021) discovered that mass vaccination reduced the risk of COVID-19-related deaths by 72% and 84% from 14 to 20 days and from 21 to 27 days after the first dose, respectively [8]. Haas et al. (2021) revealed that after the second dose, the fatality risk decreased by 96 · 7% [9]. Other studies on vaccination campaigns in Israel have examined their effects on the incidence of SARS-CoV-2 infections [10-13]. In addition, a simulation study projected that COVID-19 vaccination programmes in the United States would reduce COVID-19 deaths by 69 · 3% [14]. Overall, the extent to which the observed reduction in COVID-19 mortality is attributable to vaccination programmes around the world remains unclear. To our knowledge, this is the first study to investigate from a global perspective the health benefits of vaccination efforts.

The current study investigated the association of vaccination with COVID-19 case fatality ratio (CFR) by using observational data from 90 countries. Thus, the present study complements simulation-based research and fills the gap between clinical trial-based and real-world evidence. In addition, numerous countries have struggled to deliver vaccines and vaccinate people [15]. Vaccine hesitancy [16,17], implementation bottlenecks [18,19], and unequitable access to vaccines [20] have been identified as obstacles to achieving herd immunity to COVID-19. Our sample showed that as of 20 April, less than 8% of the world populations were vaccinated. Therefore, determining how far the world is from transforming efficacious vaccines into desired health outcomes is imperative. The current study provides a preliminary assessment and sheds light on the prospect of minimising COVID-19 deaths worldwide.

Countries vary greatly in their governments’ effectiveness and ability to implement nationwide programs. Our previous study demonstrated that countries with greater government effectiveness had lower COVID-19 CFRs [21]. In the current study, we considered a wide range of country-specific characteristics and further examined whether the associations of vaccinations with CFRs vary among countries. The results herein may assist in the identification of countries with low vaccine effectiveness and may help policy makers improve the health benefits generated by vaccination efforts.

## METHODS

### Empirical model of case fatality ratio

The purpose of investigation is to identify across countries the association of vaccine coverage with COVID-19 CFR. The sample was a longitudinal data set of 90 countries over 25 weeks, from the first week of November 2020 to the third week of April 2021. Evidence from Israel national programme indicated that as vaccine coverage increased, the incidence of COVID-19 deaths decreased. The vaccine coverage was the key predictor in the empirical model, expressed as:


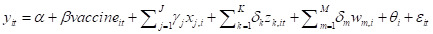
,(1)

where *y_it_* denotes the CFR for country *i* in week *t*, and *vaccine_it_* denotes the vaccine coverage for country *i* in week *t*. In this study CFR was calculated as total deaths attributed to COVID-19 per 100 confirmed cases, where both the numerator and denominator were cumulated counts since the COVID-19 outbreak in country *i* until week *t*. The focus is on a disease modification endpoint, that is, whether a COVID-19 vaccine prevents deaths among people who have been vaccinated and nonetheless become infected [18]. Vaccine coverage was measured by the number of people who received at least one vaccine dose per 10 people in the total population.

*x_j,i_* denotes time-invariant country characteristic *j* (1, ..., *J*) for country *i*, eg, government effectiveness. *z_k,it_* refers to time-varying nonpharmaceutical intervention *k* (1, …, *K*) for country *i* at week *t*; *w_m,i_* denotes continent *m* (1, …, *M*) in which county *i* is located. Continent indicators were used to capture factors that vary according to geographical location such as temperature and cultural differences. *θ_i_* is a country-specific factor affecting CFR and unobservable to analysts, such as the timeliness of data reporting. In Equation (1), *θ_i_+ε_it_* is the composite error term; *ε_it_* is the usual error term and uncorrelated with *θ_i_* or any covariates.

This study transformed CFR into logs to make the data conform more closely to the normal distribution and to improve the model fit. Therefore, the approximate change in CFR associated with one more vaccinated person is estimated by (100×β)%. This study treated *θ_i_* as random and applied random-effects model, primarily because the assumption of country fixed effects precluded us from incorporating time-invariant country characteristics in the model. In fact, both fixed- and random-effects models yielded very similar results; see Sensitivity Analysis.

### Data collection and study sample

The data used in this study were collected on 25 April 2021 from four open-access databases: Our World in Data (OW) [3,22], Worldwide Governance Indicators (WGI) [23], the Oxford COVID-19 Government Response Tracker (OxCGRT) [24] and World Development Indicators (WDI) [25].

We included countries with data in all four databases and excluded countries or regions that met the following criteria: (a) fewer than 1 million populations; (b) fewer than 25 confirmed deaths as of 18 April 2021; (c) missing data in the OxCGRT database for more than 30 days, and (d) missing data on vaccine coverage and the number of tests for COVID-19 in the OWID database during the entire study period. We transformed daily data into weekly data, and treated 1 January 2020 as the first day of the week and defined the following weeks consecutively. The first week of our study period was October 28-November 3, 2020 (Week 1), and the last week was April 14-20, 2021 (Week 25). The final sample consisted of 90 countries, or 2200 country-week observations. These countries had approximately 6.4 billion people, accounting for 83% of the world population in 2020.

**Figure 1:**
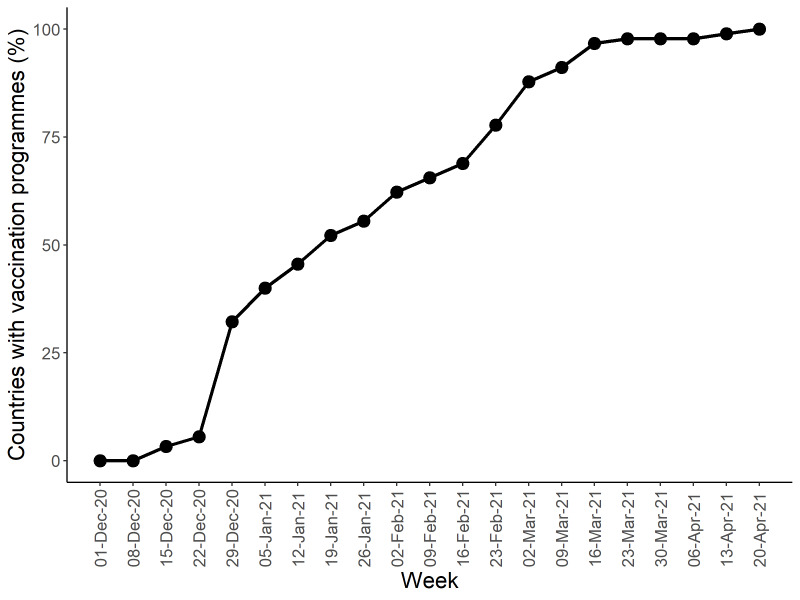
Trend for the percentage of countries with a vaccination programme. This figure presents the percentage of sample countries with a vaccination programme on a weekly basis since December 2020. The earliest week of vaccination was recorded as 9-15 December 2020. As of 20 April 2021, all 90 countries introduced COVID-19 vaccination programmes.

**Figure 2:**
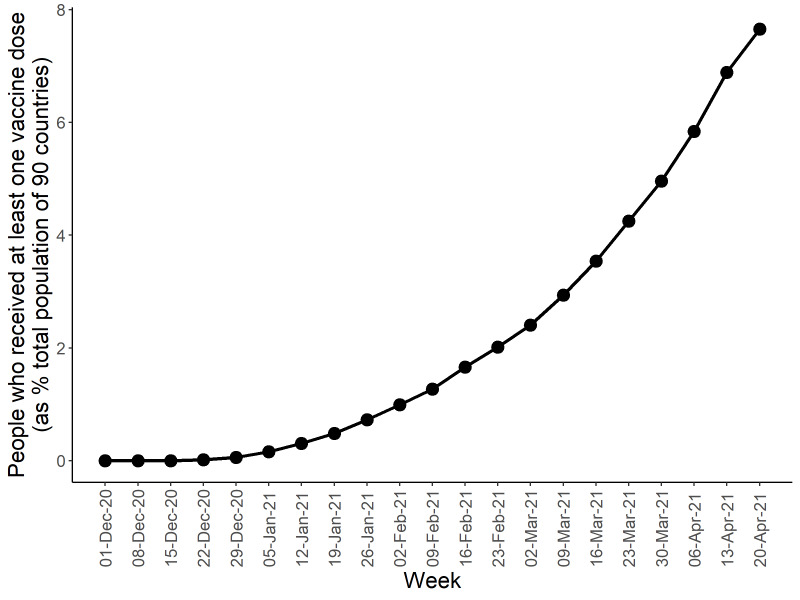
Trend for the percentage of the world population (90 countries) that was vaccinated. This figure presents the trend of the vaccine coverage measured as the number of people who received at least one vaccine dose per 100 people in the total population of 90 countries. As of 20 April 2021, approximately 7.7% of the populations were vaccinated. Those countries had approximately 6.4 billion people, accounting for 83% of the world population in 2020.

**Figure 1** presents the upward trend in the percentage of countries that implemented vaccination programmes, with the earliest week of vaccination recorded as December 9-15, 2020. **Figure 2** shows the upward trend in the percentage of people who received at least one vaccine dose among 90 countries’ total populations. During January 20-26, 2021 (Week 13 in this study), this percentage reached 0.5%. Thus the study period covered 12 weeks before and after this specific week. As of April 20, 2021 (end of Week 25), all 90 countries had a vaccination programme in place; however, only 7.7% of populations in those countries were vaccinated. Data used to produce **Figure 1** and **Figure 2** are provided in Table S1 in the **Online Supplementary Document**. Appendix S2 in the **Online Supplementary Document** provides a summary of the countries studied.

### Variable for country characteristics

The variable for government effectiveness was retrieved from the WGI database. It measures ‘the quality of public services, the quality of the civil service and the degree of its independence from political pressures, the quality of policy formulation and implementation, and the credibility of the government’s commitment to such policies’ (in page 4 of the citing paper) [26]. The government effectiveness score ranges from -2.5 to 2.5; a higher score indicates greater effectiveness.

The variables for other country characteristics included in this study were the quality of the trade and transport-related infrastructure (1 = low to 5 = high), the percentage of the population aged 65 years or older (%), the number of hospital beds per 1000 people, and the log of the gross domestic product (GDP) per capita adjusted according to purchasing power parity (in current international dollars). All of these variables were retrieved from the WDI database, except the variable for hospital beds, which was retrieved from the OWID database due to its better data quality. The most recent available year for data on country characteristics until 2019 was used. Our previous study discovered that quality of transport infrastructure was positively correlated with COVID-19 CFRs [21]. The percentage of old people and number of hospital beds were included to control for cross-country variation in demographics and health system capacity, respectively. GDP per capita was used as a proxy for a country’s socioeconomic and technological development.

### Variables for nonpharmaceutical interventions

In addition to vaccination, nonpharmaceutical interventions may affect CFRs. We included in the model government response stringency index, time to containment policy, and total tests for COVID-19 per 100 people. The stringency index is a composite score measuring the intensity of nine nonpharmaceutical interventions: school closures, workplace closures, cancellation of public events, restrictions on gathering size, closures of public transport, stay-at-home requirements, restrictions on internal movement, restrictions on international travel, and coordinated public information campaigns. The index was recorded daily for individual countries in the OxCGRT database [27]. The value of the index ranges from 0 to 100, with a higher score indicating a more stringent policy. We took the weekly mean of the stringency scores.

The time to containment policy was calculated as the number of weeks that elapsed between the date of the first confirmed death and the date of implementation of the first containment policy. We focused on the eight nonpharmaceutical interventions described previously, excluding public information campaigns. This variable was calculated retrospectively and fixed for individual countries. The conjecture is that the earlier the implementation of containment measures, the lower the CFR.

Data for total tests for COVID-19 per 100 people was retrieved from OWID. In addition, to control for the changing health care burden, we included the total confirmed cases of COVID-19 per 100 people in the previous week. The variable was lagged one period to avoid contemporaneous effect of vaccination on the incidence of infections.

### Empirical estimation

Variable for vaccine coverage and number of tests had missing values for some weeks across sample countries. To tackle the problem, we linearly interpolated both variables on weeks by using Stata command *ipolate*. In addition, to make the magnitude of coefficients more consistent, we multiplied government effectiveness scores, transport infrastructure quality index and the log of GDP per capita by 10, and divided government response stringency index by 10. The standard errors were clustered at the country level to allow for intra-country correlation. All estimations were performed using Stata 16 software (Stata Corp Inc., Texas, USA).

### Subgroup analysis by country characteristics

We further examined whether the association of vaccination with CFR varied with country characteristics. Three characteristics that appeared to be statistically significant in the regression (Equation (1)) were selected, namely, government effectiveness, transport infrastructure quality and the percentage of population aged 65 or older. In conducting subgroup analyses, we ranked countries according to their characteristics and categorised them into three groups (high, medium and low) with an equal size. The interaction terms between the vaccine coverage and the binary indicators for the country groups were then created and included in Equation (1). See Table S2 in the **Online Supplementary Document** for the classifications of the country groups.

### Analysis of dynamic relationships between vaccine coverage and case fatality ratio

As the implementation period of vaccination programmes became longer, the vaccine coverage would increase; this may lead to a greater reduction in the CFR due to accumulated protection effects among the total population. To investigate the dynamic effects, we categorized vaccine coverage (*vaccine_it_*) into nine groups according to the following thresholds: 0, <0.05, <0.1, <0.2, <0.4, <0.8, <1.6, <3.2 and ≥3.2. For example, group 9 means that the number of vaccinated people was 3.2 or more per 10 people in the population for country *i* in week *t*. Our hypothesis is that the greater the vaccine coverage, the greater the reduction in CFR. We created a binary indicator for each group and replaced *vaccine_it_* .with these indicators in Equation (1).

## Results

### Descriptive statistics

**Table 1** summarises model variables based on the 90 studied countries. The data required to calculate CFR and vaccine coverage were both retrieved from the OWID database. Country characteristics data were collected from WGI and WDI. Data for nonpharmaceutical interventions were collected from OxCGRT.

**Table 1:** Descriptive statistics of model variables*

Variable	Mean	SD	Min	Max
**Case fatality ratio:**				
Total deaths attributed to COVID-19 per 100 confirmed cases	2.08	1.38	0.05	9.87
**Vaccine coverage:**				
Total number of people who received at least one vaccine dose per 10 people in the total population	0.30	0.75	0.00	6.20
**Country characteristics**				
Government effectiveness score (-2.5 to 2.5)†	0.36	0.86	-1.34	2.22
Transport infrastructure quality index (1-5)‡	2.97	0.66	1.82	4.37
Population aged 65 or older (%)	11.61	6.81	1.16	28.00
Hospital beds per 1000 population	3.29	2.63	0.30	13.05
Gross domestic product per capita (log)	9.84	1.03	6.97	11.49
**Nonpharmaceutical interventions:**				
Government response stringency index (0-100)§	61.94	14.07	19.44	88.89
Time to containment policy since first death (weeks)	-4.56	4.83	-26.86	1.00
Total tests for COVID-19 per 100 people	39.07	60.77	0.33	583.20
Total confirmed cases of COVID-19 per 100 people	2.44	2.34	0.00	11.16
**Continent:**				
Africa	0.14	0.34	0.00	1.00
Asia	0.28	0.45	0.00	1.00
Europe	0.35	0.48	0.00	1.00
North America	0.11	0.32	0.00	1.00
South America	0.10	0.30	0.00	1.00
Oceania	0.02	0.15	0.00	1.00

SD – standard deviation

*Note: The sample had 90 countries and 2200 country-week observations.

†Government effectiveness score measures the quality of public and civil services, the quality of policy formulation and implementation, and the credibility of the government’s commitment to such policies (2.5 = greatest effectiveness).

‡Transport infrastructure quality index measures the quality of the trade and transport infrastructure (5 = highest quality).

§Government response stringency index measures the intensity of nine containment policies: school closures, workplace closures, cancellation of public events, restrictions on gathering size, closures of public transport, stay-at-home requirements, restrictions on internal movement, restrictions on international travel, and coordinated public information campaigns (100 = strictest response).

The COVID-19 CFR ranged from 0.05% to 9.87%, with a mean value of 2.08%. The mean vaccine coverage was 0.3, meaning that 0.3 people received at least one vaccine dose per 10 people in the population. Vaccine coverage varied greatly across countries; Israel had the greatest coverage of 6.2 per 10 people. The mean value of time to containment policy was negative because most countries introduced containment policies with varying degrees of intensity before the first reported death.

### Association between vaccine coverage and the case fatality ratio

The results of Equation (1) are presented in **Table 2**. The key finding was that an increase in one vaccinated person per 10 people in the population, or a 10% increase in the vaccine coverage, reduced the CFR by approximately 7.6% (95 CI = -12.6 to -2.7%, *P* = 0.002). An increase of government effectiveness score by 0.1 reduced the CFR by 7.6% (95 CI = -10.2 to -4.9%, *P* < 0.001). An increase of transport infrastructure quality index by 0.1 increased the CFR by 7.5% (95 CI = 3.8 to 11.3%, *P* < 0.001). An increase in population aged 65 or older by 1% increased the CFR by 5.4% (95 CI = 0.8 to 10.0%, *P* = 0.022). Moreover, we discovered that GDP per capita was negatively correlated with the CFR (95 CI =  7.3 to -1.0%, *P* = 0.009). Time to containment policy (95 CI = 1.0 to 8.0%, *P* = 0.012) and tests for COVID-19 (95 CI = 0.0 to 0.3%, *P* = 0.045) were positively correlated with the CFR.

**Table 2:** Results from random-effects regression for COVID-19 case fatality ratio (log)*

Predictors	Coef.	Std. Err.	(95% CI)	*P*-value
Vaccine coverage	-0.076	0.025	(-0.126, -0.027)	0.002
Government effectiveness score	-0.076	0.014	(-0.102, -0.049)	<0.001
Transport infrastructure quality index	0.075	0.019	(0.038, 0.113)	<0.001
Population aged 65 or older (%)	0.054	0.024	(0.008, 0.100)	0.022
Hospital beds per 1000 population	0.001	0.038	(-0.073, 0.075)	0.981
Gross domestic product per capita (log)	-0.042	0.016	(-0.073, -0.010)	0.009
Government response stringency index	-0.010	0.014	(-0.037, 0.017)	0.478
Time to containment policy (weeks)	0.045	0.018	(0.010, 0.080)	0.012
Total tests for COVID-19 per 100 people	0.001	0.001	(0.000, 0.003)	0.045
Confirmed cases per 100 people last week	0.017	0.015	(-0.013, 0.047)	0.267

*Note: The sample had 90 countries and 2200 country-week observations. The standard errors were clustered at the country level. The R-squared value was 0.51. For convenience of interpretation, we multiplied the following variables by 10: government effectiveness score, infrastructure quality index, and gross domestic product per capita (log). Thus the corresponding coefficient should be interpreted on the basis of a 0.1 incremental increase in these variables. In addition, we divided the original value of government response stringency index by 10; thus its coefficient should be interpreted on the basis of a 10 incremental increase. The model included the continent in which a country is located and country random effects.

### Results from subgroup analysis

**Figure 3:**
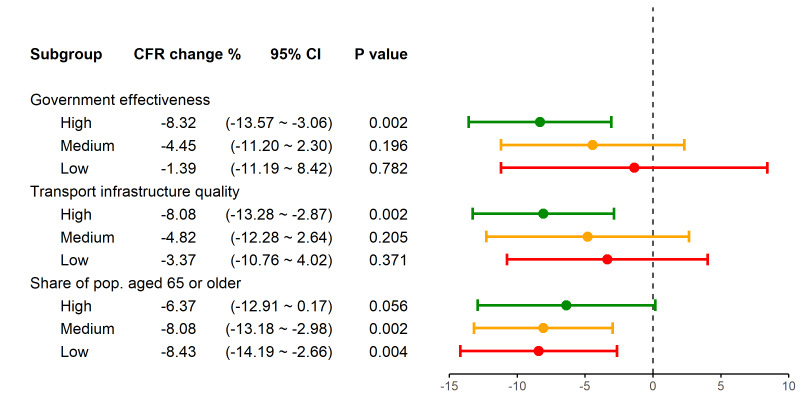
Results of subgroup analyses, by level of government effectiveness, transport infrastructure quality, and share of population aged 65 or older. For each country characteristic, countries were ranked and divided into three groups: high (green), medium (yellow), and low (red), with an equal size. The second column displays the percentage change in case fatality ratio (CFR) associated with a 10% increase in vaccine coverage, which was illustrated as the solid circle in the figure. All three regressions were conducted by using the full sample, 2200 country-week observations.

We conducted subgroup analyses according to levels of government effectiveness, quality of transport infrastructure, and percentage of old population, respectively. **Figure 3** presents the estimated coefficients of interaction terms between the vaccine coverage and the country groups. **Figure 3** shows that vaccine coverage was significantly associated with a reduced CFR only in countries with high government effectiveness (-8.3%; 95 CI = -13.6 to -3.1%, *P* = 0.002) and high-quality transportation infrastructure (-8.1%; 95 CI = -13.3 to -2.9%, *P* = 0.002). Furthermore, vaccine coverage was significantly associated with a reduced CFR in countries with a low (-8.4%; 95 CI = -14.2 to -2.7%, *P* = 0.004) and median (-8.1%; 95 CI =  13.2 to -3.0%, *P* = 0.002) percentage of old population, but not in countries with a high percentage of old population. The full regression results are presented in Appendix S3 in the **Online Supplementary Document**.

### Dynamic relationships between vaccine coverage and the case fatality ratio

**Figure 4:**
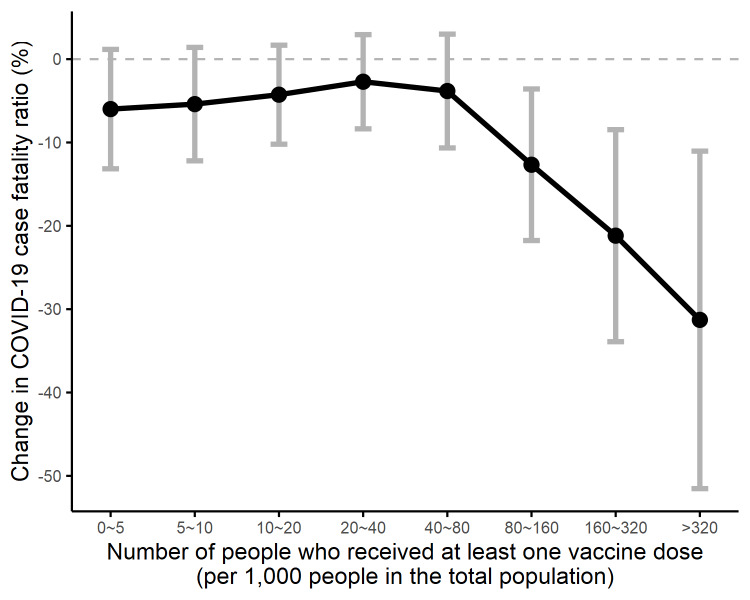
Dynamic associations between vaccine coverage and the case fatality ratio. This figure shows the percentage change in case fatality ratio (CFR) associated with different intervals of vaccine coverage. Values of CFR change on the y-axis were the estimated coefficients of binary indicator for interval of vaccine coverage provided in **Table 3**. According to **Table 3**, the reduced CFR was statistically significant when the vaccine coverage per 1000 people fell in the interval of 80-160, 160-320, and ≥320.

Analysis of dynamic relationships reveals that the vaccine coverage was associated with reduced CFRs only after the coverage reached 0.8 per 10 people. As **Table 3** shows, compared with no vaccination, when vaccine coverage achieved 0.8 to 1.6, 1.6 to 3.2 and ≥3.2 per 10 people, the CFR reduced by 12.7% (95 CI = -21.8 to -3.6%, *P* = 0.006), 21.2% (95 CI = -33.9 to -8.5%, *P* = 0.001) and 31.3% (95 CI = -51.5 to -11.0%, *P* = 0.002), respectively. This dose-dependent association is illustrated in **Figure 4**, where the x-axis specifies the vaccine coverage based on per 1000 people in the population, and the y-axis indicating the corresponding percentage change in CFR. The full regression results are presented in Appendix S4 in the **Online Supplementary Document**.

**Table 3:** Results from analysis of dynamic relationships using different intervals of vaccine coverage*

Predictors	Coef.	Std. err.	95% CI	*P* > z
Interval of vaccine coverage (No. of vaccinated people per 10 people in the population) (reference group: 0)				
<0.05	-0.060	0.037	-0.132, 0.012	0.101
0.05-0.1	-0.054	0.035	-0.122, 0.014	0.120
0.1-0.2	-0.043	0.030	-0.102, 0.017	0.159
0.2-0.4	-0.027	0.029	-0.084, 0.029	0.347
0.4-0.8	-0.038	0.035	-0.107, 0.030	0.272
0.8-1.6	-0.127	0.046	-0.218, -0.036	0.006
1.6-3.2	-0.212	0.065	-0.339, -0.085	0.001
≥3.2	-0.313	0.103	-0.515, -0.110	0.002

CI – confidence interval

*Note: The sample had 90 countries and 2200 country-week observations. Vaccine coverage was measured by the number of people received at least one vaccine dose per 10 people in the population. The standard errors were clustered at the country level. The R-squared value was 0.52. The model included country random effects and variables for country characteristics, nonpharmaceutical interventions, and continent indictors described in **Table 1****.**

### Sensitivity analysis

We conducted a series of sensitivity analysis to check if the results were robust to different model specifications. First, we changed the model assumption from country random effects to fixed effects (Appendix S5 in the **Online Supplementary Document**). Second, we used a different measure for vaccine coverage by limiting the vaccinated populations to those who received all doses prescribed by the vaccination protocol (Appendix S6 in the **Online Supplementary Document**). All of the results from sensitivity analysis are consistent with the findings reported previously.

## DISCUSSION

To our knowledge, this is the first study to systematically investigate the association between COVID-19 vaccine coverage and the case fatality ratio. Regression analysis revealed that COVID-19 vaccination was significantly associated with reduced COVID-19 CFR and that this association was stronger in countries with high government effectiveness, high-quality transportation infrastructure and younger populations. We also observed that COVID-19 vaccination was associated with CFR in a dose-dependent manner; that is, as the vaccine coverage increased, the CFR continued to decrease.

After controlling for country characteristics and nonpharmaceutical interventions, the average CFR reduction was estimated to be 7.6% for one more person with vaccination (per 10 people), or a 10% increase in vaccine coverage. Preventing death has been identified as the most important criteria for fairly distributing a COVID-19 vaccine [28]. Our results provide supporting evidence that vaccination is critical to preventing avoidable deaths from COVID-19 among infected people.

When looking the dose-dependent association, there seemed to be a required minimum coverage rate for vaccinations to take effect, which was estimated to be 8%. The sample showed that as of 20 April 2021, only 44 out of 90 countries with vaccination programmes had achieved 8% coverage. This finding may partly explain why some countries that implemented vaccination programmes saw few changes in COVID-19 deaths.

It is noteworthy that once the vaccine coverage achieved 8%, the CFR began to reduce significantly. When the vaccination programme covered more than one-third of the population, the CFR reduced by approximately 31.3% as compared with no vaccination. In our sample, seven countries (Bahrain, Chile, Hungary, Israel, United Arab Emirates, United Kingdom, United States) have vaccinated more than one-third of their populations; however, large variations existed in the vaccine coverage across countries. This suggests that a large gap remains between the observed and achievable health benefits of vaccination programmes on a global scale.

Government effectiveness is the ability of a government to create and implement high-quality public services. Therefore, government effectiveness is the intrinsic ability or preparedness of a government to implement COVID-19 vaccination programmes. In most countries, COVID-19 vaccination has been administered as a public service; the government has played the most critical role in financing, acquiring, delivering, and promoting vaccines. Our analysis revealed that improving government effectiveness is crucial because the association between vaccine coverage and CFR reductions was statically significant only for countries with high government effectiveness. For two countries that had the same vaccine coverage, the government with greater effectiveness may have greater ability to target high-risk people and allocate vaccines in a way that maximises the health benefits at the population level. Our findings are consistent with those of a simulation study that discovered that factors related to implementation contribute more to the success of vaccination programmes than does vaccine efficacy [18].

Implementation requires high logistical capacity [15]. This requirement may partly explain why the association of vaccine coverage with reduced CFR was observed only in countries with high-quality transportation infrastructure. However, we cannot be certain why vaccine coverage was associated with reduced CFRs only in countries with younger populations. One possible explanation is that many countries prioritized vaccination for old people [29]. Therefore, with the same vaccine coverage, countries with a smaller percentage of old people were more likely to have achieved a high coverage for this subgroup than countries with a high share of old people, thus leading to a greater reduction in CFR.

There are several limitations to the present study. First, our model has included a comprehensive set of control variables; however, residual confounders may exist. Second, we did not examine whether different vaccine prioritization strategies would alter the association of vaccine coverage with CFRs. Given the low vaccine coverage in many countries, identifying methods to increase the health benefits of vaccination may be important. Third, although we had overcome problems with missing data, estimation biases could still arise due to data inaccuracy. Fourth, the CFR calculated in this study is based on two assumptions: (1) the likelihood of detecting cases and deaths is consistent over time; (2) all reported cases have either recovered or died [30]. During the study period, the COVID-19 surveillance system seemed unlikely to change remarkably; thus the first assumption may hold. However, the second assumption is more difficult to verify, as data for the number of recovered patients are not provided by the databases we used. To the extent that these two assumptions are not hold, country effects assumed in Equation (1) may account for some of the resulting bias. Future studies may consider using patient-level data and applying survival analysis to tackle biases related to delayed reporting [30]. Finally, future research could reassess vaccination effectiveness by using a longer study period and taking into account the effect of virus mutations.

## CONCLUSIONS

This study evaluated the association of COVID-19 vaccination with the case fatality ratio across countries. The average CFR reduction was estimated to be 7.6% for a 10% increase in the vaccine coverage. The vaccine coverage was associated with a reduced CFR in a dose-dependent manner after the coverage reached 8% of the total population. Moreover, the reduction in CFR was stronger in countries with more effective governments, higher transport infrastructure quality, and younger populations. Vaccination programmes have yielded significant health benefits in certain countries. However, globally, a large gap remains between observed and achievable mortality reductions. It is necessary to improve the vaccine coverage and address the disparity in vaccine coverage to reduce COVID-19 fatalities around the world.

## Additional material

Online Supplementary Document
