# Supplementary material for: COVID-19 vaccinations are associated with reduced fatality rates: Evidence from cross-county quasi-experiments
Source: J Glob Health. 2021 Jul 17;11:05019. doi: 10.7189/jogh.11.05019 (PMC8285768; doi:10.7189/jogh.11.05019)
Supplement: Online Supplementary Document [file jogh-11-05019-s001.pdf]

SUPPLEMENTARY DOCUMENT

**Appendix S1**	**Trend in the percentage of countries implemented vaccination programmes and the vaccine coverage**
**Appendix S2**	**Sample countries, vaccine coverage, and rankings by government effectiveness, quality of transport infrastructure, and percentage of population aged 65 or older**
**Appendix S3**	**Regression results from subgroup analyses**
**Appendix S4**	**Regression results from analysis of dynamic relationships between vaccine coverage and the case fatality ratio**
**Appendix S5**	**Sensitivity analysis: fixed effects model**
**Appendix S6**	**Sensitivity analysis: using a different measure for vaccine coverage**

Appendix S1 Trend in the percentage of countries implemented vaccination programmes and the vaccine coverage

Table S1 Percentage of countries with COVID-19 vaccination programmes and vaccine coverage

Week	Countries with vaccination programmes (%)	People who received at least one vaccine dose (as % total population of 90 countries)
1-Dec-20	0.00	0.0000
8-Dec-20	0.00	0.0000
15-Dec-20	0.03	0.0004
22-Dec-20	0.06	0.0146
29-Dec-20	0.32	0.0611
5-Jan-21	0.40	0.1589
12-Jan-21	0.46	0.3058
19-Jan-21	0.52	0.4861
26-Jan-21	0.56	0.7250
2-Feb-21	0.62	0.9905
9-Feb-21	0.66	1.2725
16-Feb-21	0.69	1.6588
23-Feb-21	0.78	2.0150
2-Mar-21	0.88	2.4069
9-Mar-21	0.91	2.9365
16-Mar-21	0.97	3.5384
23-Mar-21	0.98	4.2497
30-Mar-21	0.98	4.9584
6-Apr-21	0.98	5.8413
13-Apr-21	0.99	6.8850
20-Apr-21	1.00	7.6538

Appendix S2 Sample countries, vaccine coverage and rankings by government effectiveness, quality of transport infrastructure, and the percentage of population aged 65 or older

Table S2 Summary of 90 countries studies

Country	Case fatality ratio	Vaccine coverage	Government effectiveness	Transport quality	Share of old people
			Ranking: 1=low, 3=high		
Albania	1.81	0.02	2	1	2
Argentina	2.18	1.24	2	2	2
Australia	3.08	0.06	3	3	2
Austria	1.67	2.03	3	3	3
Bahrain	0.36	3.69	2	2	1
Bangladesh	1.45	0.35	1	1	1
Belarus	0.71	0.22	1	1	2
Belgium	2.50	2.09	3	3	3
Bolivia	4.35	0.34	1	1	2
Bosnia and Herzegovina	4.18	0.00	1	1	3
Brazil	2.69	1.17	1	2	2
Bulgaria	3.97	0.75	2	2	3
Canada	2.07	2.53	3	3	3
Chile	2.23	4.09	3	3	2
China	4.74	0.00	2	3	2
Colombia	2.58	0.54	2	2	2
Costa Rica	1.34	0.89	2	1	2
Croatia	2.14	1.38	2	2	3
Cyprus	0.51	1.84	3	2	2
Denmark	1.01	1.93	3	3	3
Dominican Republic	1.31	0.84	1	1	1
Ecuador	4.91	0.24	1	2	2
El Salvador	3.07	0.88	1	1	2
Estonia	0.93	2.18	3	2	3
Ethiopia	1.40	0.00	1	1	1
Finland	1.06	2.43	3	3	3

Gambia	2.94	0.07	1	1	1
Germany	2.52	2.07	3	3	3
Ghana	0.84	0.27	1	1	1
Greece	3.00	1.75	2	2	3
Guatemala	3.39	0.09	1	1	1
Hungary	3.39	3.48	2	3	3
India	1.17	0.80	2	2	1
Indonesia	2.71	0.41	2	2	1
Iran	2.95	0.06	1	2	1
Iraq	1.52	0.06	1	1	1
Ireland	1.98	1.78	3	3	2
Israel	0.76	6.20	3	3	2
Italy	3.02	1.87	2	3	3
Jamaica	1.67	0.46	2	1	2
Japan	1.78	0.11	3	3	3
Jordan	1.21	0.60	2	2	1
Kazakhstan	0.95	0.40	2	2	2
Kenya	1.65	0.14	1	2	1
Kuwait	0.56	1.93	2	2	1
Latvia	1.85	1.03	3	2	3
Lithuania	1.61	2.14	3	2	3
Malawi	3.36	0.14	1	1	1
Malaysia	0.37	0.23	3	2	1
Mexico	9.22	0.88	1	2	2
Morocco	1.77	1.27	1	1	1
Mozambique	1.16	0.02	1	1	1
Myanmar	2.25	0.18	1	1	1
Netherlands	1.19	2.12	3	3	3
New Zealand	1.00	0.29	3	3	3
Norway	0.65	2.01	3	3	3
Pakistan	2.15	0.06	1	1	1
Panama	1.71	0.91	2	2	2
Paraguay	2.14	0.12	1	2	1

Peru	3.37	0.24	2	1	2
Philippines	1.69	0.12	2	2	1
Poland	2.32	1.82	2	3	3
Portugal	2.04	2.00	3	3	3
Qatar	0.20	0.35	3	3	1
Romania	2.57	1.42	1	2	3
Russia	2.24	0.71	2	2	2
Saudi Arabia	1.68	0.00	2	2	1
Serbia	0.91	2.73	2	2	3
Singapore	0.05	2.33	3	3	2
Slovak Republic	2.98	1.80	2	2	3
Slovenia	1.79	1.83	3	3	3
South Africa	3.44	0.05	2	3	1
South Korea	1.56	0.35	3	3	2
Spain	2.25	2.05	3	3	3
Sri Lanka	0.64	0.43	1	1	2
Switzerland	1.65	1.58	3	3	3
Thailand	0.24	0.09	2	2	2
Togo	0.96	0.19	1	1	1
Trinidad and Tobago	1.72	0.12	2	1	2
Tunisia	3.43	0.21	1	1	2
Turkey	0.84	1.47	2	3	2
Uganda	0.82	0.06	1	1	1
Ukraine	2.09	0.11	1	1	3
United Arab Emirates	0.31	5.14	3	3	1
United Kingdom	2.89	4.88	3	3	3
United States	1.79	3.99	3	3	3
Uruguay	1.19	3.11	3	1	2
Vietnam	1.25	0.01	2	2	2
Zambia	1.36	0.00	1	1	1
Zimbabwe	4.10	0.19	1	1	1

Note: Case fatality ratio measures the total number of deaths among COVID-19 cases. Vaccine coverage measures the number of people who received at least one vaccine dose per 10 people in the population.

Appendix S3 Regression results from subgroup analyses

Table S3.1 Results from regression of case fatality ratio (log), with interactions between vaccine coverage and indicator for high, median and low government effectiveness

Predictors	Coef.	Std. Err.	[95% CI]	P>z
Vaccine coverage				
× high government effectiveness (=1)	-0.083	0.027	[-0.136, -0.031]	0.002
× median government effectiveness (=1)	-0.045	0.034	[-0.112, 0.023]	0.196
× low government effectiveness (=1)	-0.014	0.050	[-0.112, 0.084]	0.782
Government effectiveness score	-0.075	0.013	[-0.101, -0.048]	<0.001
Transport infrastructure quality index	0.075	0.019	[0.038, 0.113]	<0.001
Population aged 65 or older (%)	0.054	0.024	[0.008, 0.100]	0.022
Hospital beds per 1,000 population	0.001	0.038	[-0.073, 0.075]	0.986
Gross domestic product per capita (log)	-0.042	0.016	[-0.073, -0.011]	0.008
Government response stringency index	-0.010	0.014	[-0.037, 0.018]	0.492
Time to containment policy (weeks)	0.045	0.018	[0.010, 0.081]	0.012
Total tests for COVID-19 per 100 people	0.001	0.001	[0.000, 0.003]	0.031
Confirmed cases per 100 people last week	0.015	0.015	[-0.016, 0.045]	0.344
Asia	-0.059	0.297	[-0.641, 0.522]	0.841
Europe	0.098	0.456	[-0.796, 0.993]	0.829
North America	0.782	0.383	[0.031, 1.534]	0.041
South America	0.602	0.359	[-0.101, 1.305]	0.093
Oceania	0.696	0.516	[-0.315, 1.707]	0.177

Note: The sample had 90 countries and 2,200 country–week observations. The standard errors were clustered at the country level. The model included country random effects.

Table S3.2 Results from regression of case fatality ratio (log), with interactions between vaccine coverage and indicator for high, median and low transport infrastructure quality

Predictors	Coef.	Std. Err.	[95% CI]	P>z
Vaccine coverage				
× high transport quality (=1)	-0.081	0.027	[-0.133, -0.029]	0.002
× median transport quality (=1)	-0.048	0.038	[-0.123, 0.026]	0.205
× low transport quality (=1)	-0.034	0.038	[-0.108, 0.040]	0.371
Government effectiveness score	-0.076	0.014	[-0.102, -0.049]	<0.001
Transport infrastructure quality index	0.076	0.019	[0.038, 0.114]	<0.001
Population aged 65 or older (%)	0.054	0.024	[0.007, 0.100]	0.023
Hospital beds per 1,000 population	0.001	0.038	[-0.073, 0.075]	0.986
Gross domestic product per capita (log)	-0.042	0.016	[-0.073, -0.010]	0.009
Government response stringency index	-0.010	0.014	[-0.038, 0.017]	0.474
Time to containment policy (weeks)	0.045	0.018	[0.010, 0.080]	0.012
Total tests for COVID-19 per 100 people	0.001	0.001	[0.000, 0.003]	0.045
Confirmed cases per 100 people last week	0.015	0.016	[-0.017, 0.046]	0.354
Asia	-0.062	0.297	[-0.643, 0.520]	0.836
Europe	0.098	0.457	[-0.797, 0.993]	0.83
North America	0.781	0.384	[0.029, 1.533]	0.042
South America	0.598	0.359	[-0.106, 1.302]	0.096
Oceania	0.694	0.516	[-0.317, 1.705]	0.178

Note: The sample had 90 countries and 2,200 country–week observations. The standard errors were clustered at the country level. The model included country random effects.

Table S3.3 Results from regression of case fatality ratio (log), with interactions between vaccine coverage and indicator for high, median and low share of population aged 65 or older

Predictors	Coef.	Std. Err.	[95% CI]	P>z
Vaccine coverage				
× high share of old people (=1)	-0.064	0.033	[-0.129, 0.002]	0.056
× median share of old people (=1)	-0.081	0.026	[-0.132, -0.030]	0.002
× low share of old people (=1)	-0.084	0.029	[-0.142, -0.027]	0.004
Government effectiveness score	-0.076	0.014	[-0.102, -0.049]	<0.001
Transport infrastructure quality index	0.075	0.019	[0.037, 0.113]	<0.001
Population aged 65 or older (%)	0.054	0.024	[0.008, 0.100]	0.023
Hospital beds per 1,000 population	0.001	0.038	[-0.073, 0.075]	0.978
Gross domestic product per capita (log)	-0.041	0.016	[-0.073, -0.010]	0.01
Government response stringency index	-0.010	0.014	[-0.037, 0.018]	0.487
Time to containment policy (weeks)	0.045	0.018	[0.010, 0.080]	0.012
Total tests for COVID-19 per 100 people	0.001	0.001	[0.000, 0.003]	0.046
Confirmed cases per 100 people last week	0.015	0.016	[-0.016, 0.046]	0.343
Asia	-0.063	0.296	[-0.644, 0.518]	0.832
Europe	0.089	0.456	[-0.804, 0.982]	0.845
North America	0.774	0.383	[0.023, 1.526]	0.043
South America	0.596	0.358	[-0.106, 1.297]	0.096
Oceania	0.697	0.515	[-0.312, 1.706]	0.176

Note: The sample had 90 countries and 2,200 country–week observations. The standard errors were clustered at the country level. The model included country random effects.

Appendix S4 Regression results from analysis of dynamic relationships between vaccine coverage and the case fatality ratio

Table S4 Results from regression of case fatality ratio (log), with intervals of vaccine coverage

Predictors	Coef.	Std. Err.	[95% CI]	P>z
Interval of vaccine coverage (no. of vaccinated people per 10 people in the population)				
(reference group: 0)				
<0.05	-0.060	0.037	[-0.132, 0.012]	0.101
0.05 – 0.1	-0.054	0.035	[-0.122, 0.014]	0.120
0.1 – 0.2	-0.043	0.030	[-0.102, 0.017]	0.159
0.2 – 0.4	-0.027	0.029	[-0.084, 0.029]	0.347
0.4 – 0.8	-0.038	0.035	[-0.107, 0.030]	0.272
0.8 – 1.6	-0.127	0.046	[-0.218, -0.036]	0.006
1.6 – 3.2	-0.212	0.065	[-0.339, -0.085]	0.001
≥3.2	-0.313	0.103	[-0.515, -0.110]	0.002
Government effectiveness score	-0.075	0.014	[-0.102, -0.049]	<0.001
Transport infrastructure quality index	0.075	0.019	[0.037, 0.113]	<0.001
Population aged 65 or older (%)	0.054	0.023	[0.008, 0.100]	0.021
Hospital beds per 1,000 population	0.003	0.037	[-0.071, 0.076]	0.944
Gross domestic product per capita (log)	-0.043	0.016	[-0.074, -0.011]	0.008
Government response stringency index	-0.006	0.013	[-0.031, 0.020]	0.676
Time to containment policy (weeks)	0.045	0.018	[0.010, 0.080]	0.013
Total tests for COVID-19 per 100 people	0.001	0.001	[0.000, 0.003]	0.043
Confirmed cases per 100 people last week	0.021	0.016	[-0.010, 0.052]	0.185
Asia	-0.060	0.300	[-0.648, 0.529]	0.843
Europe	0.091	0.458	[-0.807, 0.989]	0.842
North America	0.781	0.387	[0.023, 1.539]	0.043
South America	0.593	0.361	[-0.114, 1.301]	0.1
Oceania	0.731	0.515	[-0.277, 1.740]	0.155

Note: The sample had 90 countries and 2,200 country–week observations. The standard errors were clustered at the country level. The model included country random effects.

Appendix S5 Sensitivity analysis: fixed effects model

For robustness checks, all models discussed in the main text were re-run by using fixed-effects instead of random-effects models. Variables that did not change over time could not be estimated by the fixed-effects model and were denoted as omitted in Table E1. The results indicated that different assumptions about country effects did not change the main conclusions drawn from the current study.

Table S5.1 Results from fixed-effects regression for COVID-19 case fatality ratio (log)

Predictors	Coef.	Std. Err.	[95% CI]	P>t
Vaccine coverage	-0.077	0.025	[-0.127, -0.028]	0.003
Government effectiveness score	(omitted)			
Transport infrastructure quality index	(omitted)			
Population aged 65 or older (%)	(omitted)			
Hospital beds per 1,000 population	(omitted)			
Gross domestic product per capita (log)	(omitted)			
Government response stringency index	-0.011	0.014	[-0.039, 0.017]	0.432
Time to containment policy (weeks)	(omitted)			
Total tests for COVID-19 per 100 people	0.001	0.001	[0.000, 0.003]	0.043
Confirmed cases per 100 people last week	0.017	0.015	[-0.013, 0.048]	0.262
Asia	(omitted)			
Europe	(omitted)			
North America	(omitted)			
South America	(omitted)			
Oceania	(omitted)			

Note: The sample had 90 countries and 2,200 country–week observations. The standard errors were clustered at the country level.

Table S5.2 Results from fixed-effects regressions of case fatality ratio, with interactions between vaccine coverage and indicator for country characteristics

Predictors	Coef.	Std. Err.	[95% CI]	P>t
Vaccine coverage				
× high government effectiveness (=1)	-0.084	0.027	[-0.137, -0.031]	0.002
× median government effectiveness (=1)	-0.045	0.034	[-0.113, 0.023]	0.192
× low government effectiveness (=1)	-0.014	0.050	[-0.114, 0.086]	0.780
Vaccine coverage				
× high transport quality (=1)	-0.082	0.026	[-0.134, -0.030]	0.003
× median transport quality (=1)	-0.049	0.038	[-0.125, 0.026]	0.196
× low transport quality (=1)	-0.032	0.038	[-0.107, 0.042]	0.392
Vaccine coverage				
× high share of old people (=1)	-0.065	0.033	[-0.131, 0.001]	0.054
× median share of old people (=1)	-0.082	0.026	[-0.133, -0.031]	0.002
× low share of old people (=1)	-0.084	0.029	[-0.142, -0.026]	0.005

Note: The sample had 90 countries and 2,200 country–week observations. The standard errors were clustered at the country level. All three models included variables for country characteristics, nonpharmaceutical interventions, and continent indictors described in Table 1 in the main text.

Table S5.3 Results from fixed-effects regression of case fatality ratio (log), with intervals of vaccine coverage

Predictors	Coef.	Std. Err.	[95% CI]	P>t
Interval of vaccine coverage (no. of vaccinated people per 10 people in the population)				
(reference group: 0)				
<0.05	-0.060	0.036	[-0.133, 0.012]	0.100
0.05 – 0.1	-0.054	0.035	[-0.123, 0.014]	0.120
0.1 – 0.2	-0.043	0.030	[-0.104, 0.017]	0.156
0.2 – 0.4	-0.028	0.029	[-0.085, 0.030]	0.344
0.4 – 0.8	-0.039	0.035	[-0.109, 0.030]	0.264
0.8 – 1.6	-0.128	0.046	[-0.220, -0.036]	0.007
1.6 – 3.2	-0.214	0.065	[-0.342, -0.085]	0.001
≥3.2	-0.318	0.103	[-0.522, -0.114]	0.003

Note: The sample had 90 countries and 2,200 country–week observations. The standard errors were clustered at the country level. The model included variables for country characteristics, nonpharmaceutical interventions, and continent indictors described in Table 1 in the main text.

Appendix S6 Sensitivity analysis: using a different measure for vaccine coverage

For robustness checks, we used a different measure for vaccine coverage and re-run all models discussed in the main text. Vaccine coverage was redefined as the number of people who received all doses prescribed by the vaccination protocol per 10 people in the population. We discovered that the results remained consistent with those from the original models.

Table S6.1 Results from random-effects regression for COVID-19 case fatality ratio (log)

Predictors	Coef.	Std. Err.	[95% CI]	P>z
Vaccine coverage, restricted to fully vaccinated people	-0.069	0.032	[-0.132, -0.007]	0.030
Government effectiveness score	-0.076	0.014	[-0.103, -0.050]	<0.001
Transport infrastructure quality index	0.075	0.019	[0.038, 0.113]	0.000
Population aged 65 or older (%)	0.053	0.024	[0.007, 0.099]	0.025
Hospital beds per 1,000 population	0.001	0.038	[-0.072, 0.075]	0.972
Gross domestic product per capita (log)	-0.040	0.016	[-0.071, -0.009]	0.011
Government response stringency index	-0.010	0.014	[-0.038, 0.018]	0.466
Time to containment policy (weeks)	0.045	0.018	[0.010, 0.080]	0.012
Total tests for COVID-19 per 100 people	0.001	0.001	[0.000, 0.003]	0.096
Confirmed cases per 100 people last week	0.005	0.014	[-0.022, 0.033]	0.716
Asia	-0.070	0.297	[-0.652, 0.513]	0.814
Europe	0.111	0.457	[-0.784, 1.006]	0.808
North America	0.779	0.383	[0.029, 1.529]	0.042
South America	0.600	0.359	[-0.103, 1.303]	0.094

Oceania	0.692	0.517	[-0.320, 1.705]	0.180

Note: The sample had 90 countries and 2,200 country–week observations. The standard errors were clustered at the country level.

Table S6.2 Results from random-effects regressions of case fatality ratio, with interactions between vaccine coverage and indicator for country characteristics

Predictors	Coef.	Std. Err.	[95% CI]	P>z
Vaccine coverage restricted to fully vaccinated people				
× high government effectiveness (=1)	-0.073	0.035	[-0.143, -0.004]	0.039
× median government effectiveness (=1)	-0.046	0.047	[-0.139, 0.046]	0.328
× low government effectiveness (=1)	-0.029	0.051	[-0.130, 0.071]	0.567
Vaccine coverage restricted to fully vaccinated people				
× high transport quality (=1)	-0.074	0.035	[-0.142, -0.006]	0.033
× median transport quality (=1)	-0.033	0.050	[-0.131, 0.065]	0.514
× low transport quality (=1)	-0.014	0.047	[-0.107, 0.078]	0.764
Vaccine coverage restricted to fully vaccinated people				
× high share of old people (=1)	-0.051	0.063	[-0.176, 0.073]	0.421

× median share of old people (=1)	-0.065	0.029	[-0.122, -0.008]	0.025
× low share of old people (=1)	-0.083	0.041	[-0.162, -0.003]	0.043

Note: The sample had 90 countries and 2,200 country–week observations. The standard errors were clustered at the country level. All three models included variables for country characteristics, nonpharmaceutical interventions, and continent indictors described in Table 1 in the main text.

Table S6.3 Results from random-effects regression of case fatality ratio (log), with intervals of vaccine coverage

Predictors	Coef.	Std. Err.	[95% CI]	P>z
Interval of vaccine coverage, restricted to fully vaccinated people (reference group: 0)				
<0.05	-0.036	0.032	[-0.099, 0.026]	0.253
0.05 – 0.1	-0.040	0.029	[-0.097, 0.017]	0.168
0.1 – 0.2	-0.052	0.038	[-0.127, 0.023]	0.177
0.2 – 0.4	-0.021	0.039	[-0.097, 0.055]	0.595
0.4 – 0.8	-0.092	0.056	[-0.202, 0.018]	0.100
0.8 – 1.6	-0.158	0.066	[-0.287, -0.030]	0.016
1.6 – 3.2	-0.212	0.077	[-0.364, -0.060]	0.006
≥3.2	-0.304	0.130	[-0.558, -0.050]	0.019

Note: The sample had 90 countries and 2,200 country–week observations. The standard errors were clustered at the country level. The model included country random effects. The model included variables for country characteristics,

nonpharmaceutical interventions, and continent indictors described in Table 1 in the main text.
